# Elimination of the formation of biofilm in industrial pipes using enzyme cleaning technique

**DOI:** 10.1016/j.mex.2014.08.008

**Published:** 2014-08-26

**Authors:** Xiaobo Liu, Bo Tang, Qiuya Gu, Xiaobin Yu

**Affiliations:** The Key Laboratory of Industrial Biotechnology, Ministry of Education, School of Biotechnology, Jiangnan University, Wuxi 214122, Jiangsu, China

**Keywords:** Biofilm, Enzyme cleaning, Biofilm-forming bacteria, CIP (cleaning in place)

## Abstract

Currently, there is a growing demand in how to eliminate the biofilm formed in industrial pipelines, especially in food, fermentation, and water treatment industry. However, the traditional techniques for CIP (cleaning in place) are usually ineffective, superficial, halfway, and do not clean or sterilize microbes located in the inner layers of the biofilm. A recent strategy for removing the biofilm in pipes is employing enzymes to clean it in the circulating water system under an optimal condition. However, how to operate and control the whole cleaning process is difficult. Here, we will introduce the strategy of enzyme cleaning to make it more appropriated and effective.

• A modification of CIP method is proposed for higher efficiency by using N-acetylmuramide glycanohydrolase as catalysts whose optimal pH and temperature is 10 ± 1 and 45 ± 2 °C, respectively.
• The initial efficiency of enzyme cleaning was evaluated by testing the content of ATP in water sample using Clean-Trace™ (3M Corporation).
• Lastly, the terminal water was tested with SLYM-BART™ (HACH Corporation) to find out whether there were biofilm-forming bacteria, such as *Pseudomonas aeruginosa* (Lakretz et al. (2011) [1]), *Pseudomonas fluorescens* (O’Toole and Kolter (1998) [2]), iron bacterium, etc.

## Graphical abstract

**Figure:**
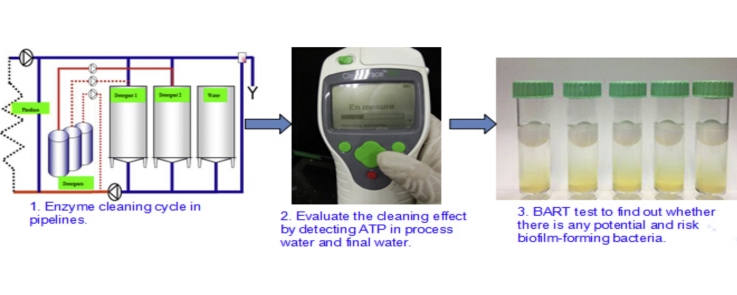

## Method details

**Fig. 1:**
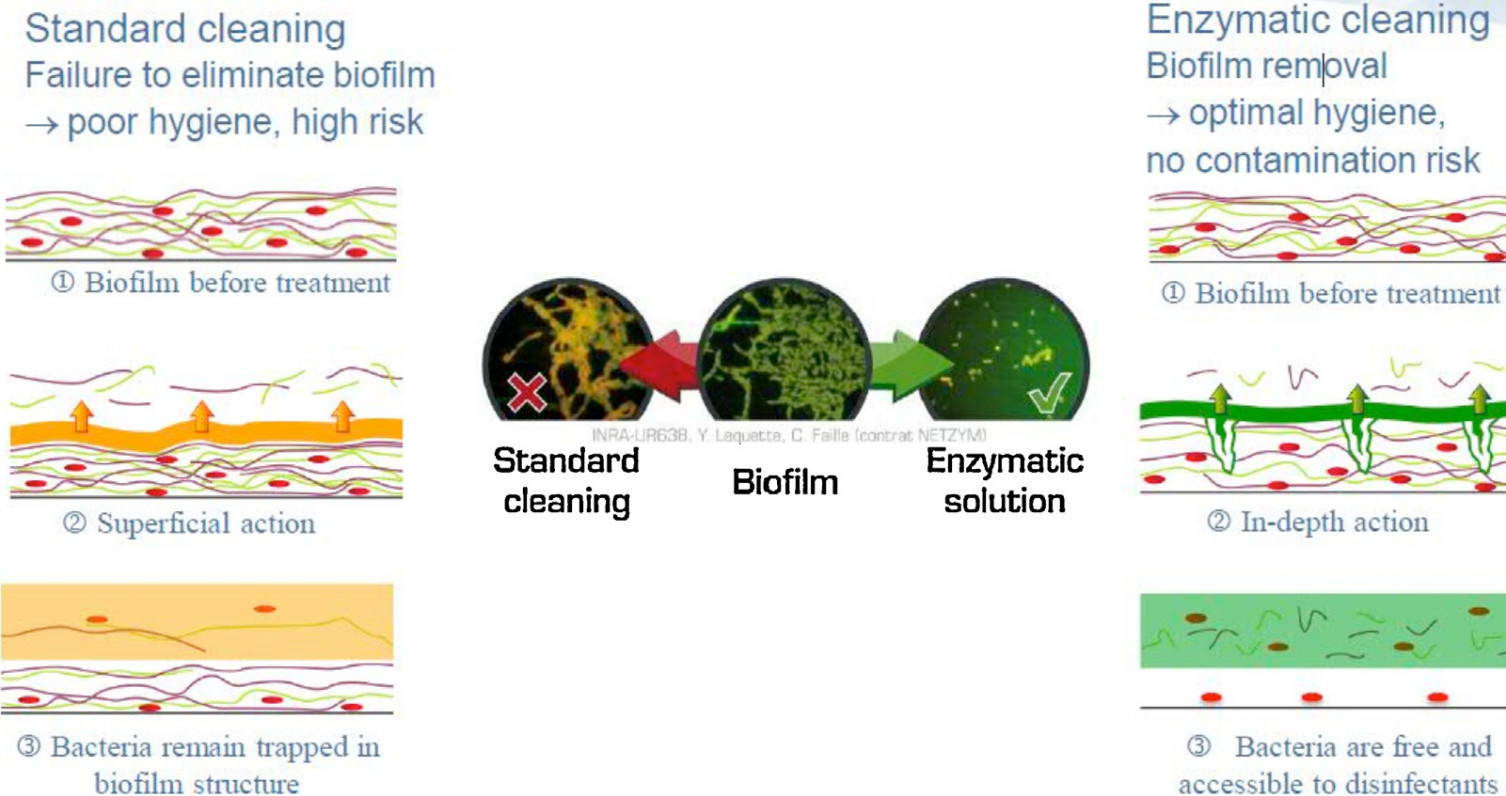
Comparison of effectiveness between the traditional CIP and enzyme cleaning technique.

In the water treatment process, traditional CIP techniques can usually remove or sterilize microbes on the surface of pipes. Taking the advantages of low cost and low energy consumption, these strategies were universally used in food, fermentation, and water treatment industry [1,3]. However, when the biofilm forms in pipelines, the traditional methods would not be available to eliminate it completely [2]. By contrast, the strategy of using muramidase to remove the biofilm in pipes is more effective and in-depth. The comparison of effectiveness between the traditional CIP and enzyme cleaning technique is shown in Fig. 1 and Table 1.

### Preparation of material

In this new strategy, N-acetylmuramide glycanohydrolase is introduced as the critical enzyme which will react with the polymeric matrix of the biofilm, reduce its adherence and make the biofilm detach from the surface. In this study, the optimal pH and temperature for reaction is 10 ± 1 and 45 ± 2 °C, respectively. The temperature of 45 ± 2 °C is used throughout the whole application procedure. The material was processed in the following manner.

### Chemicals

• Pure soda (200 g/L solution)
• Pure acid (5 mol/L HNO_3_/H_2_SO_4_/citric acid)
• 0.25% Enzyme (Biorem A1, Biorem 10)
• Aller test kit (MERCK Corporation)
• Bart test kit (HACH Corporation)
• ATP 1G kit (3M Corporation)
• 75% Alcohol

### Instruments and tools

• Incubator
• pH meter
• 500 mL sterile bottle
• Sampling bottle, flasks
• Pipette (0.1–1 mL, 5 mL)/disposable syringes (1 mL, 5 mL)

### Preparation of enzyme solution for cleaning

**Fig. 2:**
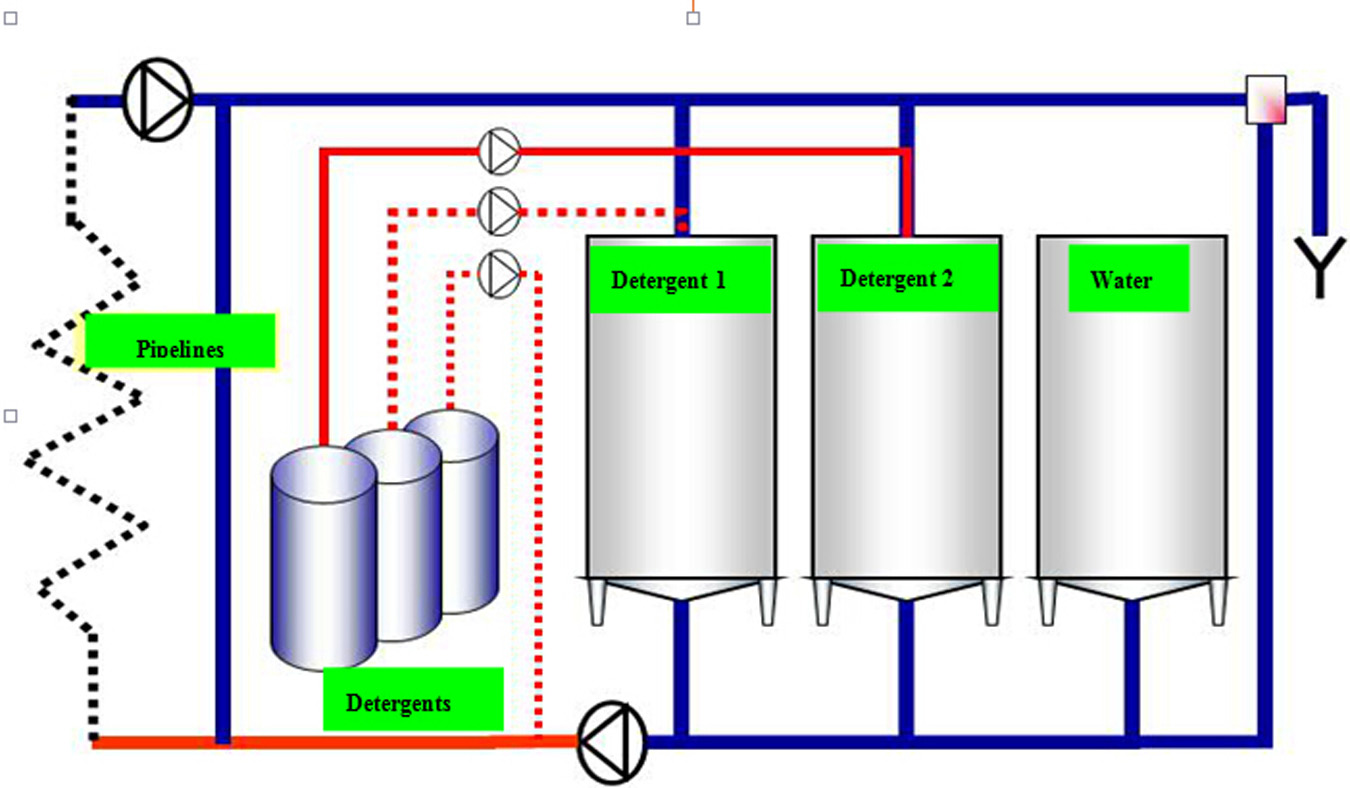
The flow chart of enzyme cleaning process.

In Fig. 2, the detergent 1 is the initial enzyme solution, and the detergent 2 represents soda or acid which were used to adjust the pH and clean the pipes. The cleaning process can be proceeded with according to the following steps:

1. Drain the retention water in all pipes and equipment.
2. Remove the in-line micro filter (*d* < 50 μm) unless they are necessary to protect equipment or be cleaned.
3. Pump detergent 2 (200 g/L soda) into water tank to keep the pH at 10 ± 1.
4. Pump detergent 1 (enzyme) into water tank to make the enzyme concentration reach 0.25%.

**Fig. 3:**
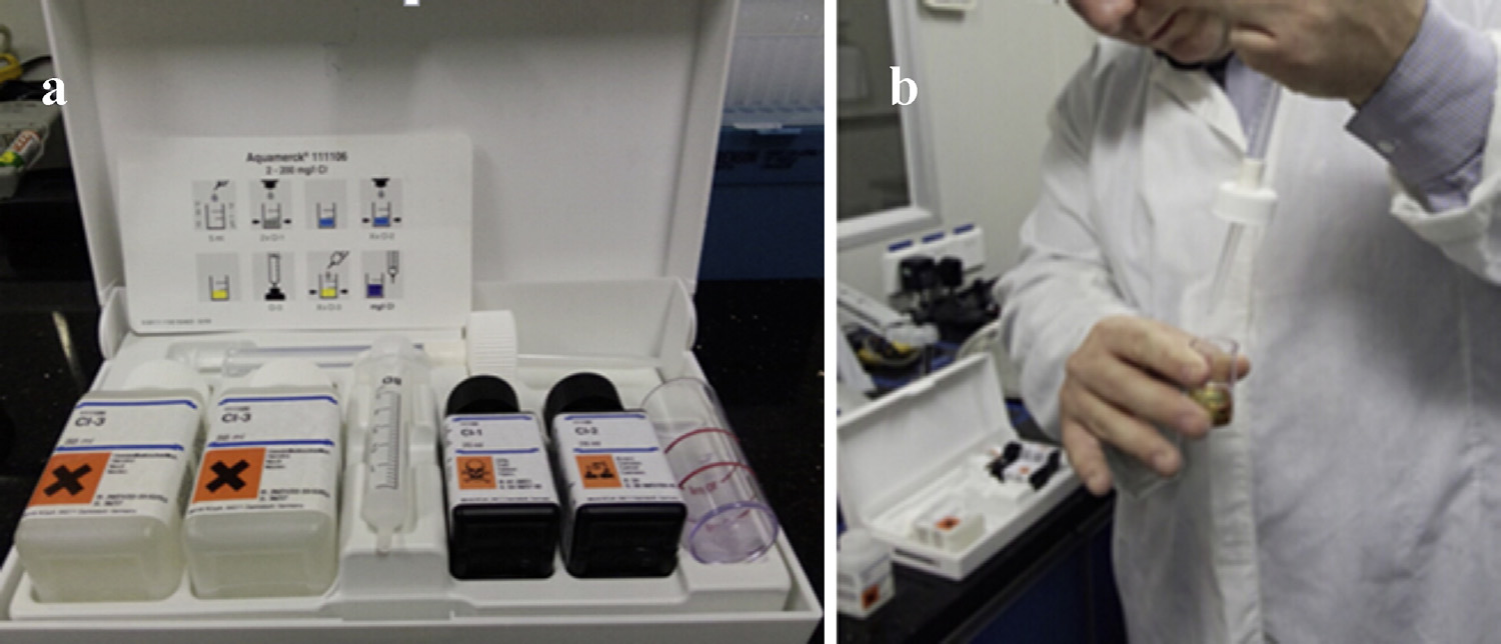
(a) Aller test kit. (b) Operation of titration experiment using the Aller test kit.

In this step, we need to test whether the concentration of enzyme solution meets the cleaning requirement using Aller test kit (Fig. 3a). Firstly, sample 500 mL enzyme solution, and then get 5 mL sample into flask using a disposable syringe. Please note that both flask and syringe need to be washed using samples previously. Add two drops of Cl-1 reagent into flask, and the sample will change into purple. Then, drop Cl-2 reagent into flask slowly and shake it simultaneously until the sample turned from purple to yellow. After that, drop Cl-3 reagent into flask slowly and shake it simultaneously until the sample changed from yellow to purple, and record the consumed volume of Cl-3 reagent as *V*_1_ (Fig. 3b). The initial water was also tested according to the above process. The concentration of enzyme solution could be calculated following the formula below:

$$E_{c}=\frac{V_{1}-V_{0}}{372}$$

Note: (a) *V*_1_ represents the volume of Cl-3 reagent consumed by enzyme solution. (b) *V*_0_ represents the volume of Cl-3 reagent consumed by the initial water. (c) *E*_*c*_ represents the concentration of enzyme solution which should be within the range of 0.25 ± 0.02%. (d) The number 372 indicated the coefficient of volume conversion from Cl-3 reagent to enzyme solution in the formula.

5. Lastly, circulate the prepared enzyme solution in all pipes and equipment which need to be cleaned.

### Test of ATP in process water

**Fig. 4:**
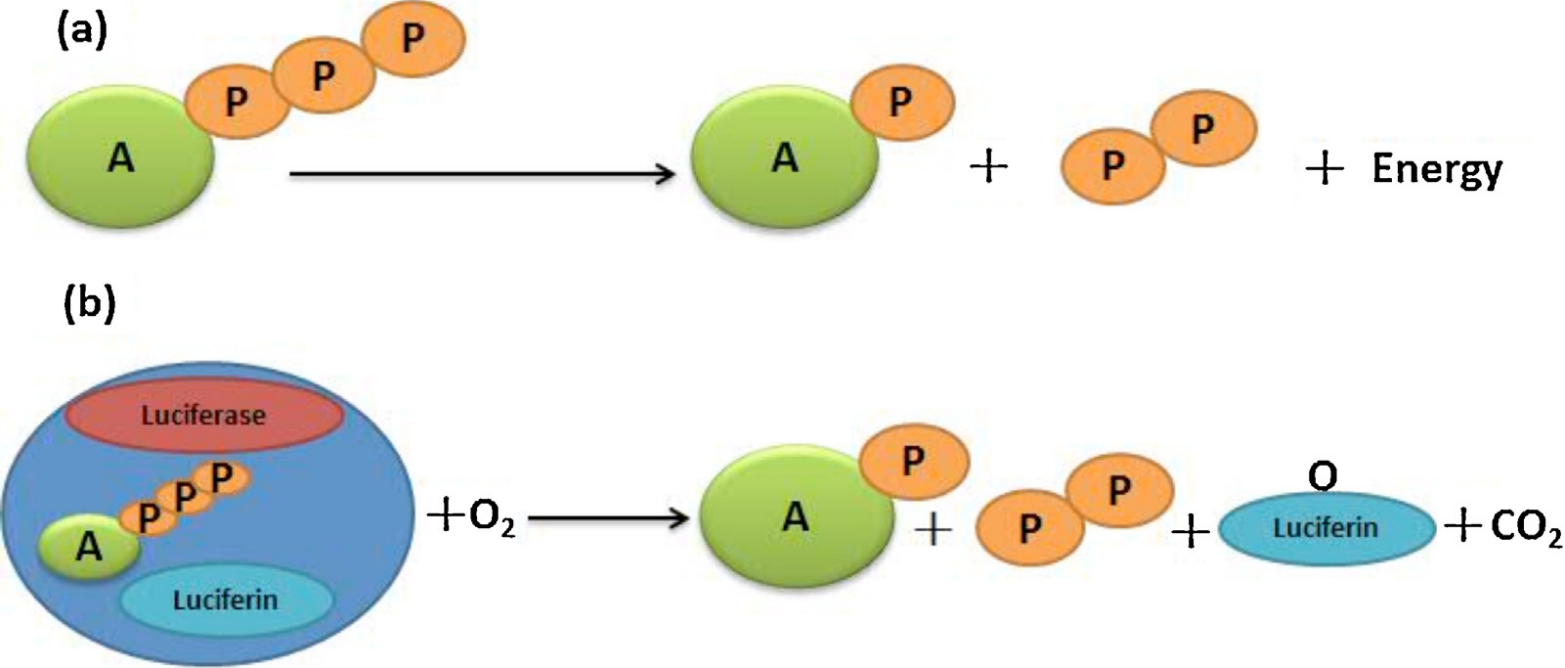
(a) Releasing energy reaction of ATP in organisms. (b) Fluorescence reaction of ATP when catalyzed by luciferase. “P” represents the phosphate group. This reaction can produce a lot of fluorescence light.

ATP is the essential energy molecule which universally exists in all organisms. Therefore, one can quickly judge whether there are any living organisms or the biofilm was completely eliminated by testing the amount of ATP (<10 cATP) in water (Fig. 4a). Here, we introduce ATP luciferin test technique which is based on the positive relationship between fluorescence intensity and ATP concentration while the fluorescence reaction is catalyzed by luciferase (Fig. 4b).

**Fig. 5:**
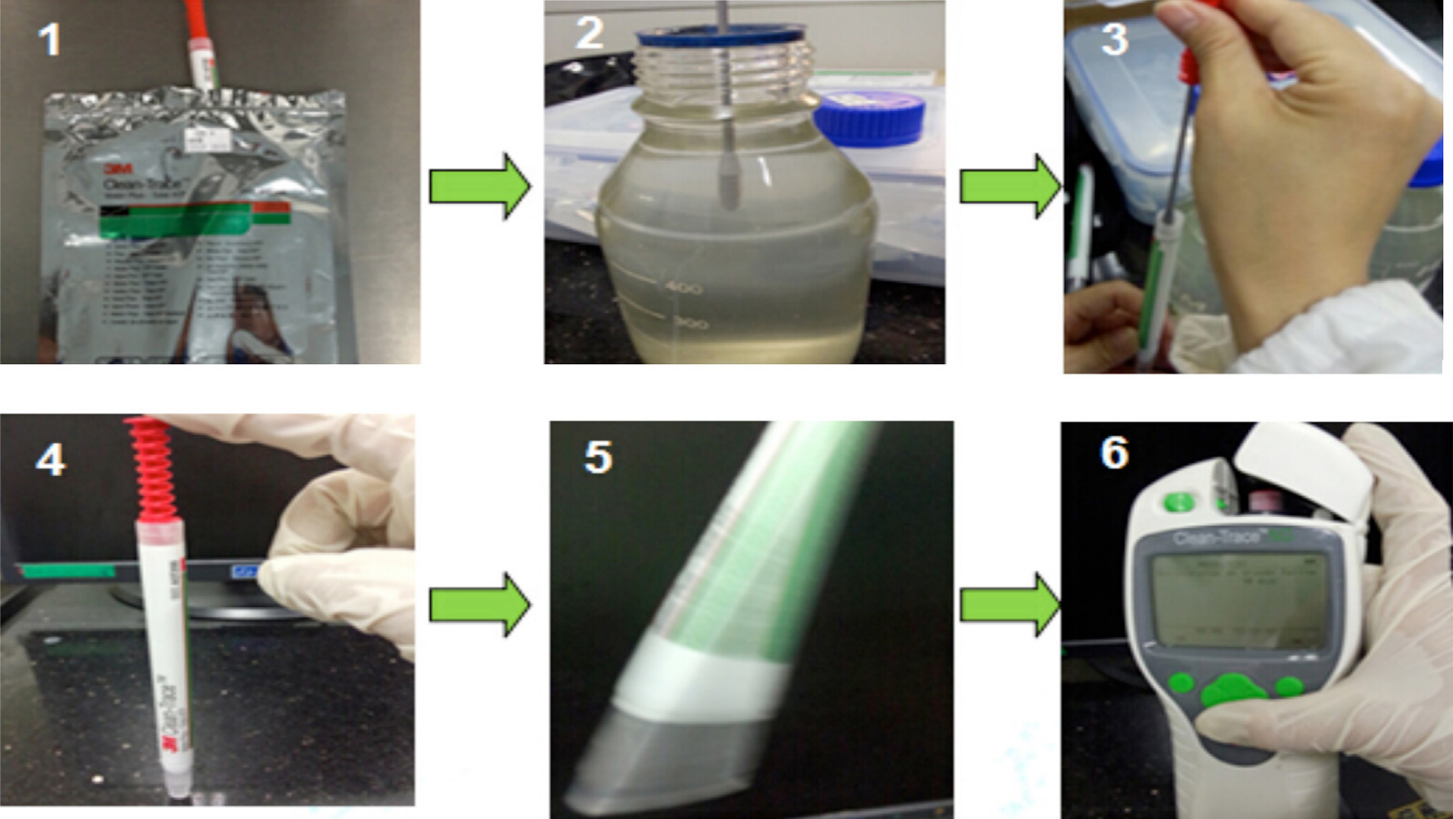
The operation steps of ATP test using Clean-Trace™ ATP detector.

The AIP test can be carried out by the following steps (shown in Fig. 5):

1. Get a water quality sampling rod out of the bag (storage at 2–8 °C) and keep it in balance for 10 min at room temperature before use.
2. Pull rod core and immerse it into the sample for a few seconds, then take it out and ensure there is a drop of liquid at the bottom of the rod core.
3. Carefully insert the rod core back into test bars (make sure the core do not recline on wall and the sample rods can enter into the reaction liquid at the bottom). Then press the red part of the sample rods to make it fully inserted into the reaction liquid.
4. Shake the test bar for 5 s to mix the liquid more completely.
5. Turn on the Clean-Trace™ ATP detector. When self-check process is completed, open the slot and insert test bar.
6. Lastly, close the slot and start to test for about 10 s, record the results (unit: RLU). For enzyme cleaning, if the result is below 100 RLU, the cleaning effect is good, otherwise it should continue.

### Bart test for biofilm-forming bacteria

The SLYM-BARTs can be used as a P/A test capable of indicating to some extent the possible organisms present in the water sample. Slime-forming bacteria are able to produce copious amounts of slime without necessarily having to use any iron. Iron bacteria also produce slime but usually it is thinner and involves the accumulation of various forms of iron (http://65.87.233.94/BARTs/SLYM.html?).

**Fig. 6:**
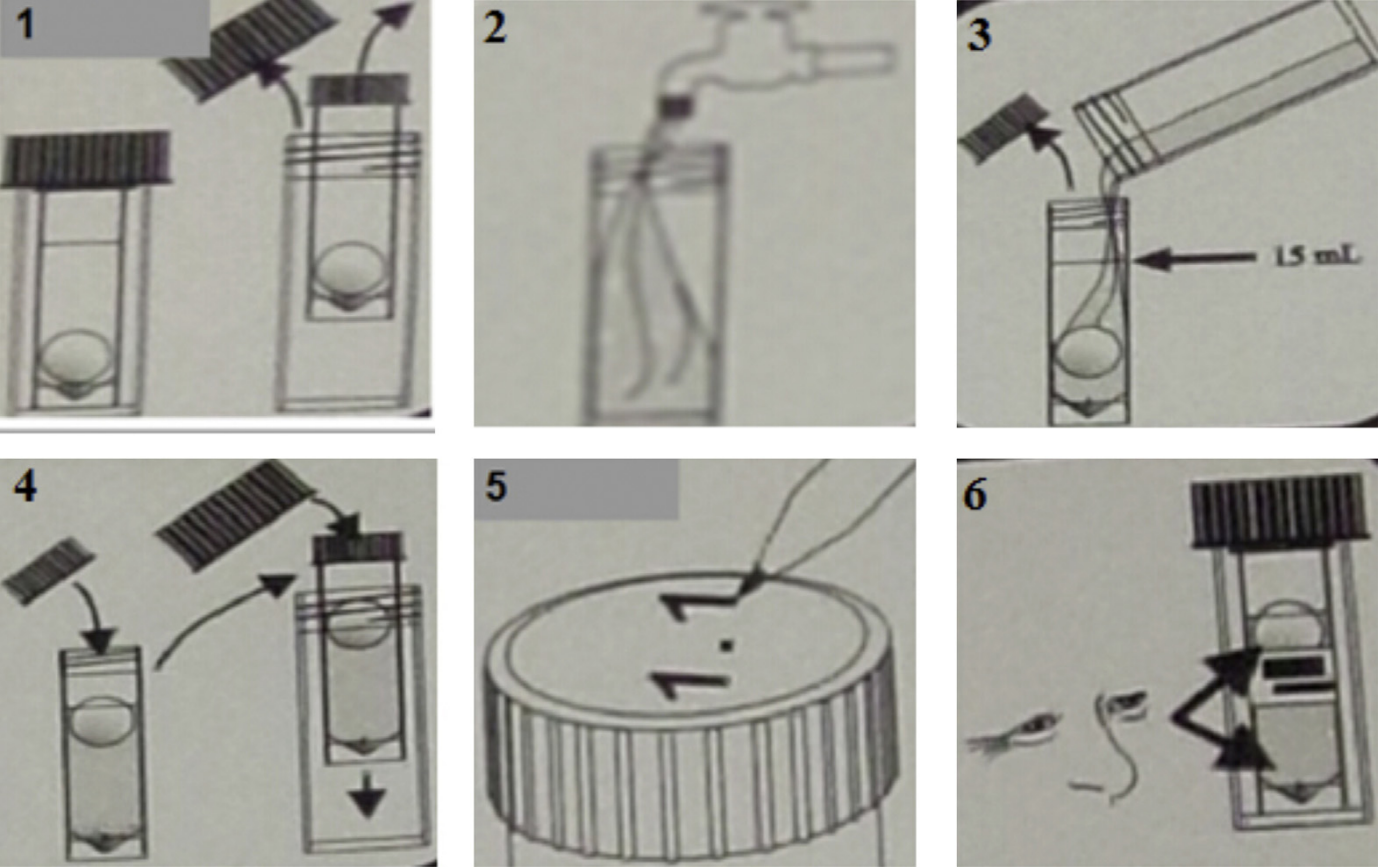
The operation steps of BART test for slime-forming bacteria.

After enzyme cleaning, one should test the water samples again, to assess the efficacy of the procedure. If the BARTs reveal there are still biofilm-forming bacteria, enzyme cleaning should be carried out again. The slime-forming bacteria should be tested to evaluate the micro-ecology of the pipelines after enzyme cleaning (see Table 1). The potentially slime-forming bacteria can be tested following the steps below (Fig. 6):

1. Remove the inner tube from the outer tube.
2. Using the outer tube from the BART, or a different aseptic container, collect at least 20 mL of sample. Note: Do not touch or contaminate the inside of the tube or lid. Use aseptic technique.
3. Fill the inner tuber with sample until the level reaches the fill line. Note: After removing the cap from the inner tube, set it down directly on a *clean surface*. To avoid contamination, do not invert the cap.
4. Tightly screw the cap back on the inner tube. Return the inner tube to the outer tube and screw the outer cap on tightly. Allow the ball to rise at its own speed. “*Do not shake or swirl the tube*”.
5. Label the outer tube with the date and sample origin.
6. Place the BART tube away from direct sunlight and allow to be incubated at room temperature. Check the BART visually for reaction daily.

**Fig. 7:**
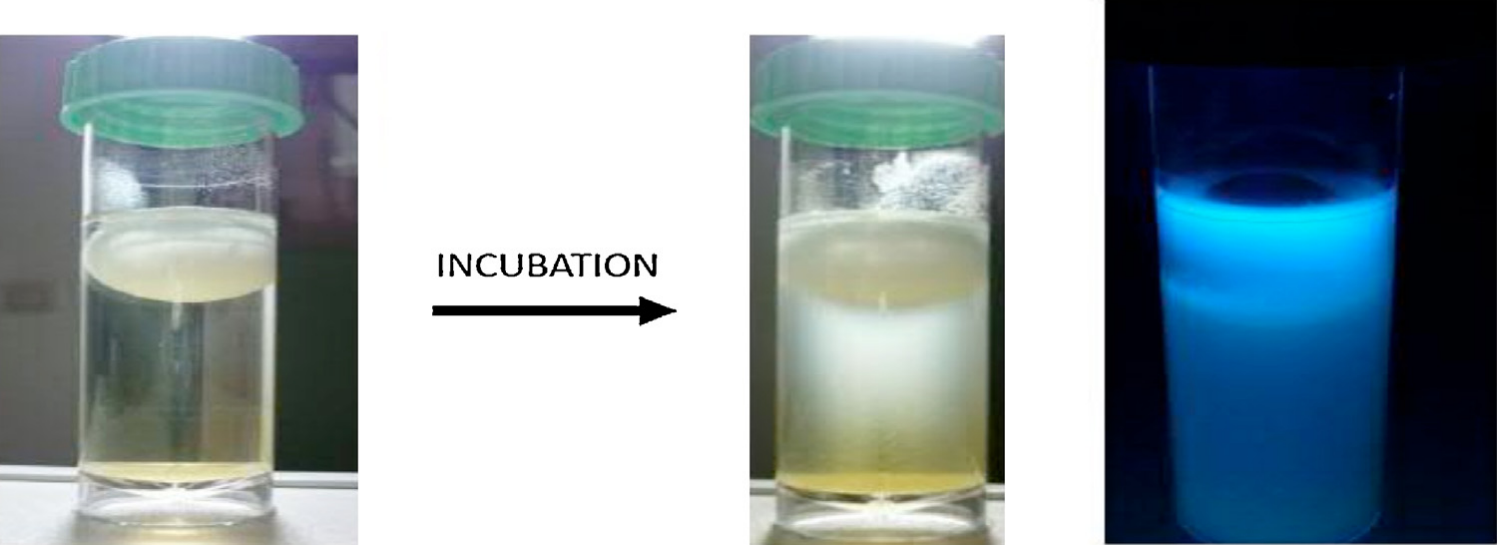
The growth of slime-forming bacteria in BART tubes after incubation. The fluorescence in tubes was observed under the UV light.

Slime-forming bacteria generally produce the thickest slime formations under aerobic (oxidative) conditions, which develop around the floating ball. Growth may be recognized as a cloudy or gel-like growth, which can be localized or occur throughout the sample. These growths are usually white, gray, yellow, or beige in color and can darken over time (http://65.87.233.94/BARTs/SLYM.html?). Meanwhile, they can usually have fluorescence when incubated in BART (Fig. 7). For these samples, safely dispose using a dedicated microwave oven or by autoclaving them.

## Figures and Tables

**Table 1:** Comparison of the effectiveness between enzyme cleaning technique and traditional CIP methods.

Samples	Traditional CIP technique		Enzyme cleaning technique	
	cATP(RLU)	SLYM-BARTs	cATP(RLU)	SLYM-BARTs
Raw water	160	−	120	−
Sand-filtered water	286	+	100	−
Carbon-filtered water	612	++	84	−
RO water	1246	+++	90	+
Deoxygenated water	878	+++	76	−

RLU value indicates the concentration of ATP (cATP) in the water sample. For enzyme cleaning, if the result is below 100 RLU, the cleaning effect is good, otherwise it should continue. “−” represents no slime-forming bacteria are detected in this water sample. “+” indicates slime-forming bacteria are occasionally found in this water sample. “++” indicates slime-forming bacteria obviously exist in this water sample. “+++” indicates many slime-forming bacteria can be detected easily in this water sample.
